# Correction: Ultrafast conversion of carcinogenic 4-nitrophenol into 4-aminophenol in the dark catalyzed by surface interaction on BiPO_4_/g-C_3_N_4_ nanostructures in the presence of NaBH_4_

**DOI:** 10.1039/d1ra90144c

**Published:** 2021-09-15

**Authors:** Ahmed B. Azzam, Ridha Djellabi, Sheta M. Sheta, S. M. El-Sheikh

**Affiliations:** Faculty of Science, Chemistry Department, Helwan University Ain Helwan Cairo 11795 Egypt ahmed_azzam2000@hotmail.com +201285259709; Università degli Studi di Milano, Dip. Chimica and INSTM-UdR Milano Via Golgi, 19 20133 Milano Italy; Department of Inorganic Chemistry, National Research Centre 33, El-Behouth St., Dokki Giza 12622 Egypt; Nanomaterials and Nanotechnology Department, Advanced Materials Division, Central Metallurgical R & D Institute (CMRDI) P. O. Box, 87 Helwan 11421 Cairo Egypt

## Abstract

Correction for ‘Ultrafast conversion of carcinogenic 4-nitrophenol into 4-aminophenol in the dark catalyzed by surface interaction on BiPO_4_/g-C_3_N_4_ nanostructures in the presence of NaBH_4_’ by Ahmed B. Azzam *et al.*, *RSC Adv.*, 2021, **11**, 18797–18808. DOI: 10.1039/D1RA02852A.

The authors regret that some misleading statements were included in section 3.2.1 ‘Effect of initial concentration on 4-NP’. The corrected version of section 3.2.1 is presented below. There are no changes to Fig. 8 or its caption.

**Fig. 8:**
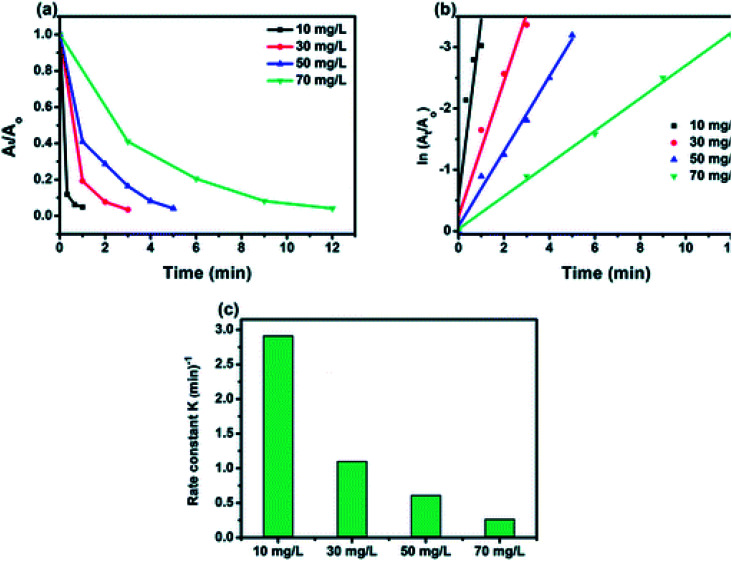
Effect of initial concentration on reduction rate of 4-NP (a); corresponding linear transform ln(*A*_*t*_/*A*_0_) = *f*(*t*) of the 4-NP reduction kinetics curves (b); comparison of the rate constant value for the reduction of the 4-NP over initial different concentration (c). ([4-NP] = 10–70 mg L^−1^, [catalyst] = 0.5 g L^−1^).

**3.2.1 Effect of initial concentration of 4-NP**

The effect of the initial concentration of 4-NP on the catalytic efficiency rate using 50% BiPO_4_/g-C_3_N_4_ catalyst was carried out by varying the concentration from 10 to 70 mg L^−1^, and the obtained results are shown in Fig. 8a. Interestingly, 50% BiPO_4_/g-C_3_N_4_ was able to reduce all 4-NP solutions at concentrations from 10 to 70 mg L^−1^, reflecting the high efficiency of such a catalyst towards this 4-NP reduction. At lower concentrations, a superior constant rate was recorded due to the availability of a large number of catalytic sites per given amount of 4-NP moles. And *vice versa*, the higher the concentration, the lower the rate constant (Fig. 8b), due to the high competition of 4-NP molecules on the limited sites. In addition, the number of molecules adsorbed at the surface of the BiPO_4_/g-C_3_N_4_ heterojunction increases with the increase in concentration of 4-nitrophenol and hence, the surface becomes saturated by 4-nitrophenol molecules. This leads to a decrease in concentration of BH_4_^−^ ions approaching the surface of the BiPO_4_/g-C_3_N_4_ heterojunction, hence lowering the rate of hydrogen transfer from BH_4_^−^ ion to the 4-nitrophenol molecule.

The Royal Society of Chemistry apologises for these errors and any consequent inconvenience to authors and readers.

## Supplementary Material

